# Recurrence Score^®^ Result Impacts Treatment Decisions in Hormone Receptor-Positive, HER2-Negative Patients with Early Breast Cancer in a Real-World Setting—Results of the IRMA Trial

**DOI:** 10.3390/cancers14215365

**Published:** 2022-10-31

**Authors:** Dominik Dannehl, Tobias Engler, Lea L. Volmer, Annette Staebler, Anna K. Fischer, Martin Weiss, Markus Hahn, Christina B. Walter, Eva-Maria Grischke, Falko Fend, Florin-Andrei Taran, Sara Y. Brucker, Andreas D. Hartkopf

**Affiliations:** 1 Department for Womens’ Health, Tuebingen University, 72076 Tübingen, Germany; 2 Department for Pathology and Neuropathology, Tuebingen University, 72076 Tübingen, Germany; 3 Department for Gynecology and Obstetrics, Freiburg University, 79085 Freiburg im Breisgau, Germany; 4 Department for Gynecology and Obstetrics, Ulm University, 89081 Ulm, Germany

**Keywords:** oncotype DX, recurrence score, breast cancer, individualized therapy

## Abstract

**Simple Summary:**

Hormone receptor-positive (HR+), HER2-negative (HER2−) is the most common breast cancer subtype (approximately 75% of all breast cancer cases). Adjuvant chemotherapy can be administered to patients that undergo operative tumor removal with only few metastatic axillary lymph nodes (0–3). However, using classical risk biomarkers to guide adjuvant chemotherapy recommendation leads to overtreatment of patients including unnecessary possible chemotherapy-related toxicities. This prospective study assessed whether the multigene-expression assay Oncotype DX^®^ that has been validated in two large clinical phase III trials, effectively reduces adjuvant chemotherapy recommendation in a real-world setting. This study could demonstrate that absolute adjuvant chemotherapy recommendation can be reduced by nearly 15% using Oncotype DX^®^. Furthermore, this study could show that the Oncotype DX^®^ recurrence score correlates to classic biomarkers that are commonly used to classify the aggressiveness of breast cancer.

**Abstract:**

Background: Patients with hormone receptor-positive (HR+), HER2-negative (HER2−) early breast cancer (eBC) with a high risk of relapse often undergo adjuvant chemotherapy. However, only a few patients will gain benefit from chemotherapy. Since classical tumor characteristics (grade, tumor size, lymph node involvement, and Ki67) are of limited value to predict chemotherapy efficacy, multigene expression assays such as the Oncotype DX^®^ test were developed to reduce over- and undertreatment. The IRMA trial analyzed the impact of Recurrence Score^®^ (RS) assessment on adjuvant treatment recommendations. Materials and methods: The RS result was assessed in patients with HR+/HER2− unilateral eBC with 0–3 pathologic lymph nodes who underwent primary surgical treatment at the Department for Women’s Health of Tuebingen University, Germany. Therapy recommendations without knowledge of the RS result were compared to therapy recommendations with awareness of the RS result. Results: In total, 245 patients underwent RS assessment. Without knowledge of the RS result, 92/245 patients (37.6%) would have been advised to receive chemotherapy. After RS assessment, 56/245 patients (22.9%) were advised to undergo chemotherapy. Chemotherapy was waived in 47/92 patients (51.1%) that were initially recommended to receive it. Chemotherapy was added in 11/153 patients (7.2%) that were recommended to not receive it initially. Summary: Using the RS result to guide adjuvant treatment decisions in HR+/HER2− breast cancer led to a substantial reduction of chemotherapy. In view of the results achieved in prospective studies, the RS result is among other risk-factors suitable for the individualization of adjuvant systemic therapy.

## 1. Introduction

Breast cancer is the most common cancer in women in Germany and worldwide [1,2]. The most frequent tumor subtype is hormone receptor-positive (HR+), HER2-negative (HER2−) early breast cancer (eBC). Patients with no or 1–3 involved pathologic lymph nodes account for approximately 70% of all breast cancer cases [3,4]. Patients with high clinicopathologic risk factors, such as large tumor size, high tumor grade, lymph node involvement, or a high proliferative index (Ki67) often undergo chemotherapy to reduce the risk of recurrence [5,6]. Yet, many of these patients may not benefit from chemotherapy. Hence, recent research has focused on biomarkers that can predict chemotherapy benefit in eBC, and several multigene-expression assays have been developed and validated in large prospective phase III trials [7,8,9,10,11].

One of the various commercially available multigene-expression assays is the Oncotype DX^®^ test. It analyzes the expression pattern of 16 breast cancer-related genes and 5 reference genes to calculate a Recurrence Score^®^ (RS) result, ranging from 1 to 100, to identify patients at a high risk of recurrence [12]. Retrospective analyses of biomaterial from the prospective NSABP B-20 (lymph node negative) and SWOG-8814 (lymph node positive) studies were able to demonstrate that patients with a high Recurrence Score (RS > 30) result are likely to benefit from chemotherapy [13,14,15]. The prospective randomized TAILORx clinical trial subsequently found that endocrine therapy is non-inferior to chemoendocrine therapy in node negative patients with an RS 11–25 [10]. In node-positive patients (RxPONDER trial), however, only postmenopausal women with an RS < 26 did not benefit from chemotherapy [11].

The IRMA (impact of Recurrence Score on adjuvant treatment decisions and tumor cell dissemination in estrogen receptor-positive and HER2-negative patients with early breast cancer) trial was designed to prospectively evaluate the impact of RS testing on adjuvant therapy recommendations in a clinical real-world setting. The primary endpoint was to evaluate the change in adjuvant chemotherapy recommendation after RS testing as compared to chemotherapy recommendation without knowledge of the RS result. Secondary endpoints were the influence of the RS result on tumor cell dissemination (which will be reported elsewhere), and to assess the association of the RS result with clinicopathologic factors.

## 2. Materials and Methods

IRMA is a prospective, single-center investigator-initiated registry study. It was conducted according to the guidelines of the Declaration of Helsinki and approved by the Ethics Committee of Tuebingen University (789/2018BO2). Furthermore, the study was registered under the ID NCT03961880. The study was supported by Exact Sciences.

All patients included in this analysis were treated for eBC at the Department for Women’s Health of Tuebingen University Hospital, Germany. Only patients with HR+/HER2− unilateral eBC without extensive lymph-node involvement (0–3 positive lymph nodes) who underwent complete surgical resection at the Department for Women’s Health of Tubingen University were eligible for this study. To facilitate decision making, enrollment into the IRMA study could be based on clinical lymph node status. Exclusion criteria were primary systemic therapy, recurrent or metastatic disease, bilateral breast cancer, or a previous history of secondary malignancy.

Tumors were counted as HR+ if they had a positive estrogen receptor (ER) and/or a positive progesterone receptor (PR) expression according to immunohistochemistry (≥10% positive cells for ER, ≥10% positive cells for PR). The HER2-status was assessed to local standards by using the HERCEPT test (DAKO, Glostrup, Denmark). Expression of HER2 was scored on a 0 to +3 scale. Tumors with a score of +3 were considered HER2-positive. In case of a score of +2, HER2 amplification was determined by fluorescence in-situ hybridization using the Pathvysion^®^ Kit (Vysis, Downers Grove, IL, USA). Ki67 was assessed using the M7240 monoclonal mouse anti-human Ki67 antibody MIB-1 (Agilent Dako, Santa Clara, CA, USA). The number of Ki67 positive cell nuclei was estimated for the entire core biopsy in a semiquantitative evaluation in steps of 10% by a board certified pathologist as part of the clinical routine workup. Based on St. Gallen consensus for breast cancer, Ki67 values were divided into two prognostic groups: 0–19% (Ki67 low) and ≥20% (Ki67 high) [16]. For Oncotype DX analyses, paraffin-embedded tumor tissue samples were submitted to Exact Sciences (Redwood City, CA, USA), according to guidelines provided by the manufacturer. Based on the classification that was used in TAILORx, patients were divided into two prognostic groups: 0–25 (RS low) and ≥26 (RS high) [10,11].

Surgery and radiation therapy were administered according to national guidelines. Postoperative systemic treatment recommendation was assessed twice: first, an interdisciplinary tumor conference at Tuebingen University Hospital advised the receipt of chemotherapy or not without knowledge of the RS results. Subsequently, in a further tumor conference after receipt of the RS result, a new decision on adjuvant chemotherapy was made.

Data processing and statistical analysis were performed using Jupyter Notebook (Version 6.3.0, Project Jupyter, open-access and community developed) on Anaconda (Version 3.0, Anaconda Inc., Austin, TX, USA) with the Python extension packages pandas (Version 1.4.1, open-access and community developed), numeric Python (Version 1.22.2, open-access and community developed), and scientific Python (Version 1.8.0, open-access and community developed). Data visualization was achieved using the Python extension packages Matplotlib (Version 3.5.0, open-access and community developed) and Plotly (Version 3.5.0, open-access and community developed). Lucid^®^ (Lucid Software Inc., South Jordan, UT, USA) was used for designing flow charts and data visualization.

Normality distribution was assessed using the Shapiro–Wilk test. Normally distributed data were tested for significance using two-sided Student’s *t*-test with a significance level of α = 0.05. Non-normally distributed data were analyzed using the Mann–Whitney-U test with a significance level of α = 0.05 as well. The relationship between nominally scaled independent variables was assessed using the *x*^2^-test.

## 3. Results

### 3.1. Patient Characteristics

In total, 245 patients were included in this study. Table 1 displays the main patient characteristics. Of all patients, 34.7% were premenopausal, whereas 65.3% were postmenopausal. Mean age (±SD) was 57.0 ± 11.3 years. The most common histology was no special type (76.7%). The most common grading was G2 (75.5%) while the most frequent tumor classifications were T1 (55.1%) and N0 (72.2%). Mean Ki67 values were 19.6 ± 12.5% and mean RS values were 16.9 ± 10.2.

### 3.2. Recurrence Score Results

A total of 14.7% of patients had an RS result ≥ 26 (Table 1). Tumor grade was associated with the RS result (*p* < 0.0001, *x*^2^-test). The most frequent grade in the RS high group was G3 (50%) and G2 (81.3%) in the RS low group. There was no association between the RS result and age, menopausal status, histology, tumor size, or lymph node involvement.

**Figure 1:**
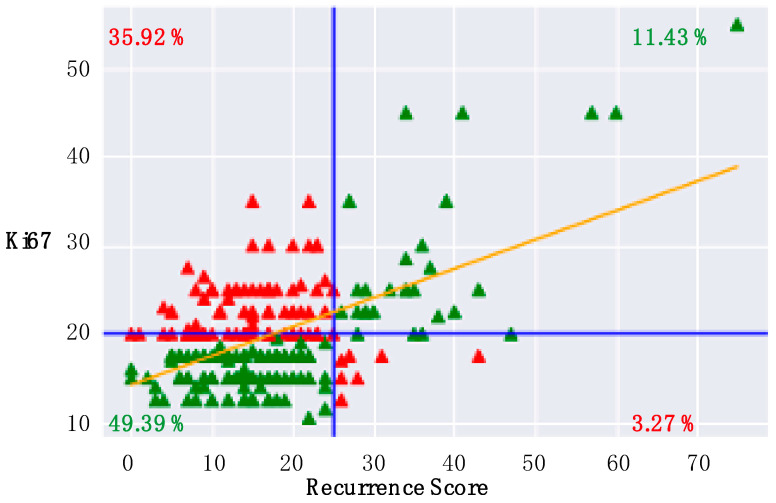
Correlation of Recurrence Score and Ki67: A clinical cut-off value for the Recurrence Score (RS) is ≥26 and for Ki67 is ≥20% (blue lines). Patients with concordant RS and Ki67 values are displayed in green. Discordant RS and Ki67 values are highlighted in red. The yellow line extrapolates the correlation coefficient (Rho = 0.54).

Patients with an RS result ≥ 26 exhibited a significantly higher mean Ki67 proliferation index (RS high vs. RS low: 31.5 ± 20.3% vs. 16.3 ± 7.6%; *p* < 0.0001, Mann–Whitney U-test). Nevertheless, a concordant classification of Ki67 and RS result in the categories “high” and “low” was found in only 60.8% of the cases (49.4% concordant “low”, 11.4% concordant “high”). In 39% of all cases a discordant classification can be observed. However, in 35.9% a low RS is associated with a high Ki67 and only in 3.3% a high RS result is associated with a low Ki67. Figure 1 displays the distribution of RS and Ki67.

## 4. Chemotherapy Recommendation

**Figure 2:**
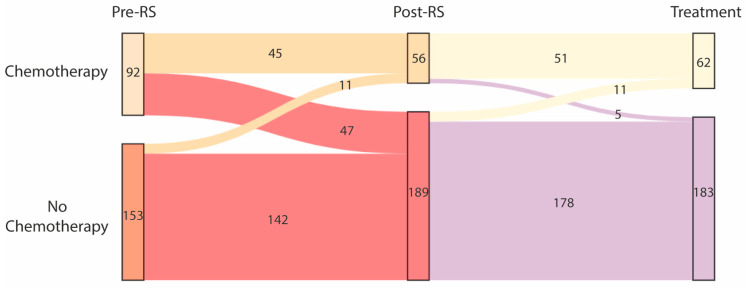
Changes in treatment recommendation due to Recurrence Score (RS) assessment and final treatment decision.

Without knowledge of the RS result, 92/245 patients (37.6%) would have received chemotherapy (Figure 2). After RS assessment, 56/245 patients (22.9%) were advised to undergo chemotherapy. Chemotherapy was waived in 47/92 patients (51.1%) that were initially recommended to receive it. Chemotherapy was added in 11/153 patients (7.2%) that were initially recommended to not receive it. Furthermore, 62/245 patients (25.3%) actually started with adjuvant chemotherapy.

Without knowledge of the Recurrence Score, 92/245 patients were recommended chemotherapy and 153/245 patients were recommended to not undergo chemotherapy (left column). After knowledge of the RS result, chemotherapy recommendation was changed in 58 patients: 56/245 patients were recommended chemotherapy and 189/245 patients were recommended to not undergo chemotherapy (middle column). After patient informed consent, 62/245 patients eventually started chemotherapy (right column).

After awareness of the RS result, 22.9% of all patients were recommended chemotherapy. Mean age of patients in the chemotherapy group was 52.2 ± 11.9 years (Table 2). These patients were significantly younger compared to patients that were not recommended to receive chemotherapy (58.5 ± 10.8 years; *p* = 0.0002, *t*-test). There was an association between menopausal status and recommendation for chemotherapy. Whereas 29.1% of patients in the no chemotherapy group were premenopausal, 53.6% of patients that were recommended to receive chemotherapy were premenopausal (*p* = 0.0013, *x*^2^-test). High tumor grade was also significantly associated with the recommendation to receive chemotherapy (*p* < 0.0001, *x*^2^-test). 41.1% of all patients in the chemotherapy group had G3 compared to 2.7% in the no chemotherapy group. Furthermore, larger tumor size significantly correlates to chemotherapy recommendation (*p* = 0.0106, *x*^2^-test). In the chemotherapy group, 60.7% of all patients had larger tumors (pT2-4) compared to 40.2% in the no chemotherapy group. Moreover, pathologic lymph node involvement was also correlated with chemotherapy recommendation (*p* = 0.0454, *x*^2^-test). The Ki67 proliferation index was significantly higher in patients that were recommended to receive chemotherapy (chemo vs. no chemo: 29.3 ± 17.7% vs. 16.7 ± 8.6%; *p* < 0.0001, Mann–Whitney U-test). The RS result was also significantly higher in patients in the chemotherapy group (chemo vs. no chemo: 29.6 ± 11.9 vs. 13.2 ± 5.7; *p* < 0.0001, Mann–Whitney U-test). Consequently, significantly more patients in the chemotherapy group were classified in the RS high group (RS ≥ 26: 64.3%; *p* < 0.0001, *x*^2^-test).

## 5. Discussion

The IRMA trial is a prospective study that demonstrates how treatment recommendations in clinical routine are impacted using multigene-expression assays. Using the RS result, chemotherapy was spared in approximately half of the patients that were recommended to receive it by means of “classical” clinicopathologic risk factors. Conversely, RS result could identify a small group of patients who might benefit from chemotherapy, although they were initially recommended to not receive it. These findings are highly comparable with the REMAR trial, a multicentric prospective trial that also aimed at characterizing changes in treatment recommendation after the use of Oncotype DX assay [17]. Both the IRMA and the REMAR trials found that using the RS result leads to a meaningful reduction of chemotherapy use and, with respect to the results of TAILORx and RxPONDER trials, can reduce overtreatment [10,11].

Multiple studies aim at assessing the influence of classical clinicopathologic risk factors such as tumor size, tumor grade, Ki67, lymph node involvement, age, ER, and PR status on the results of multigene-expression assays [8,18,19]. This information can be used to select patients that mostly benefit from the use of multigene expression assays [20,21]. The MINDACT trial validated the use of the 70-gene signature to assess a low-risk group with an excellent prognosis [8]. Patients that either had a low genomic with a high clinical risk, or a low clinical with a high genomic risk did not benefit from adjuvant chemotherapy regarding distant recurrence or OS [8]. In contrast, the TAILORx and RxPONDER trials did not answer the question whether patients with a high RS result, but a low clinical risk, can safely omit chemotherapy or whether patients with a low RS result, but a high clinical risk, would have gained benefit from chemotherapy administration. In the IRMA trial, 29/62 patients (46.8%) actually received chemotherapy with an RS result < 26 while 3/183 patients (1.6%) did not receive chemotherapy although they had an RS > 26.

Moreover, statistical models that condense clinicopathologic and genetic risk factors were developed [22]. Whereas a secondary analysis of all patients (pre- and postmenopausal) of the TAILORx trial could demonstrate that patients with an RS < 16 do not gain additional prognostic information by including clinicopathologic risk factors, premenopausal lymph node-negative patients with an RS result between 16 and 25 and a high clinicopathologic risk have increased distant recurrence rates. In particular, women younger than 50 with an RS result between 16 and 25 seem to benefit from additional chemotherapy [10,18,23]. In the IRMA trial, 19/245 (7.8%) patients aged <50 years exhibited an RS result between 16 and 25, yet 12 of these (63.2%) did not receive chemotherapy. Moreover, in the RxPONDER trial, premenopausal patients with lymph-node involvement did benefit from chemotherapy regardless of the RS result and there was no statistical association between RS result and the efficacy of chemotherapy when considering a Recurrence Score of 0–25. [11]. In our current trial, 26/245 patients were premenopausal and displayed node-positive eBC. Prior to the publication of the RxPONDER results on 9 December 2020, 5/16 premenopausal node-positive patients (31.3%) were recommended to receive chemotherapy. Yet, after publication of the RxPONDER results, 8/10 premenopausal node-positive patients (80%) were advised to receive chemotherapy.

Further studies are required to elucidate why, in contrast to postmenopausal patients, premenopausal women with high clinicopathologic risk factors do benefit from chemotherapy administration even in case of a low-risk RS result. A popular hypothesis is that chemotherapy induces ovarian function suppression in premenopausal women [24]. Although there are emerging data that the addition of ovarian function suppression to endocrine treatment positively impacts prognosis, no study has investigated whether this approach can be used to replace adjuvant chemotherapy. Moreover, retrospective analyses may suggest that RS result partially depends on the menstrual cycle, since key genes that comprise the RS algorithm are expressed differently in the different menstrual cycle phases [25].

According to recent German guidelines, multigene-expression assays can be used in HR+/HER2− patients with 0–3 involved lymph nodes if established clinical and pathological factors do not allow therapy decisions regarding the use of chemotherapy [26]. However, the IRMA trial clearly shows that even highly experienced oncologists working in a large tertiary care center are not able to correctly classify the risk of recurrence in HR+/HER2− eBC by solely using “classical” clinicopathologic risk factors. In comparison, the recent national comprehensive cancer network (NCCN) guidelines recommend that the use of multigene-expression assays should be considered in patients with HR+/HER2− eBC based on lymph node involvement. Patients with no pathologic lymph nodes involved (pN0) should be recommended to assess RS if the tumor size is larger than 0.5 cm. Patients with one to three involved pathologic lymph nodes (pN1) should be considered to undergo RS testing if they are eligible for chemotherapy administration [27].

Since additional use of multigene-expression assays implies further financial burden for the health care system, models have been developed to describe the cost-effectiveness of these tests [28,29,30]. A recently published study demonstrated that the use of multigene-expression assays in Canada could significantly reduce chemotherapy prescription in the RS low group. Interestingly, they also highlighted that RS testing is associated with excess costs in 70- to 80-year-old patients. In this cohort, chemotherapy prescription is not concordant to RS testing result [30]. Thus, the authors underlined the importance of careful patient selection in these age groups. Moreover, another recently published study, evaluating the cost-effectiveness of different multigene-expression assays in Germany, showed that all available assays (Oncotype DX, Mammaprint, Prosigna, Endopredict) reduce overall treatment costs [29]. Several statistical models using clinicopathologic risk factors to identify patients that will most likely benefit from RS testing are currently available, which might help to further reduce overall treatment costs [20,21,31].

In the absence of multigene-expression assays, clinical decision-making regarding chemotherapy use is based on the presumed molecular subtype of the tumor [32]. To classify HR+/HER2− tumors into luminal A-like and luminal B-like, most clinicians are using the proliferation marker Ki67. The International Ki67 Working Group (IKWG) reported that very low (<5%) and high values (>30%) of Ki67 are well defined cut-off values to recommend chemotherapy or not [33]. A predefined secondary analysis of the monarchE trial recently found that Ki67 values ≥ 20% are prognostic of a worse prognosis [34]. However, Ki67 assessment is prone to a high interrater variability, pointing out the need for a more standardized Ki67 assessment [33]. In line with previous studies, we found a modest correlation between RS and Ki67 results [19,35]. Using a threshold of 20%, as recently validated in the monarchE trial [34], we found a concordance rate of 60.8% as compared to the RS low and high groups. The correlation was highest among high-risk patients (high Ki67 and high RS, Rho = 0.71). Nevertheless, in 35.2% of all cases high Ki67 values were associated with low RS values, highlighting that a high Ki67 value is not a valid surrogate for the RS result. Yet, a low Ki67 value correlates with low RS values in 93.8%, suggesting that Ki67 values should be partially implemented in preclinical risk scores to assess which patient needs to undergo RS testing.

The strength of this study is its prospective design, and that the IRMA trial was incorporated into the clinical routine. Thus, the IRMA trial was able to describe the influence of RS testing on therapy recommendation in a real-world situation. The reduced deviation from therapy recommendation after awareness of the RS result compared to similar studies may be attributed to the single center interdisciplinary tumor board at a tertiary care center of the highest standard [17]. However, some patients diverge from final therapy recommendation: 8/11 patients (72.7%) that were not recommended to undergo chemotherapy after RS result, but eventually received chemotherapy, were recommended to undergo chemotherapy based on clinicopathologic risk factors. The remainder stated they wanted to receive chemotherapy due to elevated security needs. All patients (100%) that did not undergo chemotherapy albeit a high RS also would have received a chemotherapy recommendation based on clinicopathologic risk factors. However, contraindications to chemotherapy were only reported in 1/5 (20%) patients. Although the results of IRMA were similar to comparable multicentric studies, the single center approach limits external validity of IRMA [17]. Another limitation of this study is that follow-up data are not available, and it is therefore not possible to assess how clinicopathologic factors, information on the RS result, and treatment recommendations will impact survival.

## 6. Conclusions

This prospective study, which was closely related to clinical practice, showed that the use of adjuvant chemotherapy was substantially reduced by determining the RS result. Only a few patients who would not have been recommended adjuvant chemotherapy if the RS result had not been determined were recommended to receive it after obtaining the RS result. In addition to other clinicopathologic risk factors, the RS result is useful for individualizing adjuvant therapy recommendations in patients with HR+/HER2− breast carcinoma.

## Figures and Tables

**Table 1:** Overall patient characteristics.

Items	Overall	RS < 26	RS ≥ 26	*p*-Value
**Patients**	245	209	36	<0.0001
	100.0%	85.3%	14.7%	
**Age**	57.0 ± 11.3	57.5 ± 11.0	54.4 ± 12.8	0.1383
**Menopausal status**				
Premenopausal	85	70	15	0.4460
	34.7%	33.5%	41.7%	
Postmenopausal	160	139	21	
	65.3%	66.5%	58.3%	
**Histology**				
NST	188	158	30	0.5891
	76.7%	75.6%	83.3%	
ILC	49	44	5	
	20.0%	21.1%	13.9%	
Other	8	7	1	
	3.3%	3.4%	2.8%	
**Grading**				
G1	32	29	3	<0.0001
	13.1%	13.9%	8.3%	
G2	185	170	15	
	75.5%	81.3%	41.7%	
G3	28	10	18	
	11.4%	4.8%	50.0%	
**Tumor size**				
pT1	135	120	15	0.1156
	55.1%	57.4%	41.7%	
pT2-4	110	89	21	
	44.9%	42.6%	58.3%	
**Nodal involvement**				
pN0	177	153	24	0.5433
	72.2%	73.2%	66.7%	
pN1	68	56	12	
	27.8%	26.8%	33.3%	
**Ki67**	19.6 ± 12.5%	16.3 ± 7.6%	31.5 ± 20.3%	<0.0001
**RS**	16.9 ± 10.2	13.8 ± 5.8	35.4 ± 10.6	<0.0001

NST = Non-special type, ILC = Invasive lobular carcinoma, RS = Recurrence Score.

**Table 2:** Patient characteristics compared with chemotherapy recommendation in knowledge of Recurrence Score result.

Items	Overall	No Chemo	Chemo	*p*-Value
**Patients**	245	189	56	
	100.0%	77.1%	22.9%	
**Age**	57.0 ± 11.3	58.5 ± 10.8	52.2 ± 11.9	0.0002
**Menopausal status**				
Premenopausal	85	55	30	0.0013
	34.7%	29.1%	53.6%	
Postmenopausal	160	134	26	
	65.3%	70.9%	46.4%	
**Histology**				
NST	188	158	46	0.5159
	76.7%	75.1%	82.1%	
ILC	49	40	9	
	20.0%	21.2%	16.1%	
Other	8	7	1	
	3.3%	3.7%	1.8%	
**Grading**				
G1	32	28	4	<0.0001
	13.1%	14.8%	7.1%	
G2	185	156	29	
	75.5%	82.5%	41.8%	
G3	28	5	23	
	11.4%	2.7%	41.1%	
**Tumor size**				
pT1	135	113	22	0.0106
	55.1%	59.8%	39.3%	
pT2-4	110	76	34	
	44.9%	40.2%	60.7%	
**Nodal involvement**				
pN0	177	142	34	0.0454
	72.2%	75.7%	60.7%	
pN1	68	46	22	
	27.8%	24.3%	39.3%	
**Ki67**	19.6 ± 12.5%	16.7 ± 8.6%	29.3 ± 17.7%	<0.0001
**RS**	16.9 ± 10.2	13.2 ± 5.7	29.6 ± 11.9	<0.0001
**RS Group**				
Low	209	189	20	<0.0001
	85.3%	100%	35.7%	
High	36	0	36	
	14.7%	0%	64.3%	

NST = Non-special type, ILC = Invasive lobular carcinoma, RS = Recurrence Score.

## Data Availability

The data presented in this study are available on request from the corresponding author. The data are not publicly available due to the German data protection acts in respect to patient data.
